# Adolescents with a history of specific language impairment (SLI): Strengths and difficulties in social, emotional and behavioral functioning^☆^

**DOI:** 10.1016/j.ridd.2013.08.043

**Published:** 2013-11

**Authors:** Gina Conti-Ramsden, Pearl L.H. Mok, Andrew Pickles, Kevin Durkin

**Affiliations:** a School of Psychological Sciences, Communication and Deafness, The University of Manchester, Oxford Road, Manchester M13 9PL, UK; b Institute of Psychiatry, King's College London, 16 De Crespigny Park, London SE5 8AF, UK; c School of Psychological Sciences and Health, University of Strathclyde, 40 George Street, Glasgow G1 1QE, UK

**Keywords:** CAMHS, Child and Adolescent Mental Health Services, PIQ, performance IQ, SDQ, Strengths and Difficulties Questionnaire, SLI, specific language impairment, TD, typically developing, Specific language impairment (SLI), Prosocial behavior, Peer problems, Emotional symptoms, Conduct problems, Hyperactivity

## Abstract

Adolescents with specific language impairment (SLI) are at a greater risk of emotional and behavioral problems compared to their typically developing (TD) peers, but little is known about their self-perceived strengths and difficulties. In this study, the self-reported social, emotional and behavioral functioning of 139 adolescents with a history of SLI and 124 TD individuals at age 16 was examined. The self-report version of the Strengths and Difficulties Questionnaire (SDQ) was used to assess their prosocial behavior and levels of peer, emotional and behavioral difficulties. Associations of these areas of functioning with gender, verbal and non-verbal skills were also investigated. Adolescents with a history of SLI were more likely than their TD peers to report higher levels of peer problems, emotional symptoms, hyperactivity and conduct problems. The majority of adolescents in both groups (87% SLI and 96% TD), however, reported prosocial behavior within the typical range. Difficulty with peer relations was the strongest differentiator between the groups, with the odds of reporting borderline or abnormally high levels of peer problems being 12 times higher for individuals with a history of SLI. Adolescents with poorer receptive language skills were also more likely to report higher levels of emotional and behavioral difficulties. The findings of this study identify likely traits that may lead to referral to services.

## Highlights

• Adolescents with SLI report having more difficulties with peers.
• They also report having more mental health problems than do typical adolescents.
• Problematic peer relations is the strongest differentiator between the two groups.
• Poorer receptive language is related to more emotional/behavioral problems.
• Despite peer difficulties, most adolescents with SLI see themselves as prosocial.

## 1 Introduction

Adolescence is a challenging developmental period. Up to half of all adult difficulties with mental health have their onset in adolescence (Belfer, 2008). Some groups of individuals appear to be more vulnerable, particularly those with developmental disorders, such as autism spectrum disorders (ASD) and specific language impairment (SLI) (Clegg, Hollis, Mawhood, & Rutter, 2005; Simonoff et al., 2013; Snowling, Bishop, Stothard, Chipchase, & Kaplan, 2006). SLI is a common disorder affecting 5 to 7% of the population (Tomblin et al., 1997). Children with a history of SLI have difficulties learning to talk despite adequate hearing, nonverbal cognitive skills and no obvious signs of neurobiological problems. Although usually detected in early childhood, there is considerable evidence to suggest that individuals with SLI have persistent language difficulties in adolescence and into adulthood (Conti-Ramsden, St Clair, Pickles, & Durkin, 2012).

Language skills could be expected to be related to socioemotional functioning and behavioral adjustment for a number of reasons. Language supports emotional self-regulation (Cole, Armstrong, & Pemberton, 2010) and social-cognitive competence is associated with language ability (Im-Bolter, Cohen, & Farnia, 2013). Experiencing difficulties in communication makes it more difficult to relate to others (Brinton & Fujiki, 1993). Difficulties in expressing one's needs and feelings and/or understanding messages from others may incur frustration and chronic distress (Brinton & Fujiki, 2010). Consistent with these assumptions, adolescents with a history of SLI are at a greater risk of emotional and behavioral difficulties (Durkin & Conti-Ramsden, 2010; Im-Bolter & Cohen, 2007; Toppelberg & Shapiro, 2000). A recent meta-analysis indicates that young people with SLI are two times more likely to exhibit abnormal levels of emotional and behavioral difficulties than their typically developing (TD) peers (Goh & O’Kearney, 2012). They are also more likely to experience social difficulties in peer relations (St Clair, Pickles, Durkin, & Conti-Ramsden, 2011) and the development of friendships (Durkin & Conti-Ramsden, 2007).

Nevertheless, young people with SLI are sociable and have a desire to engage in social interactions with peers (Hart, Fujiki, Brinton, & Hart, 2004; Wadman, Durkin, & Conti-Ramsden, 2008). Less is known about prosocial behaviors in adolescents with SLI. Prosocial behaviors are those intended to benefit others, such as helping and sharing. There is some evidence that, in childhood, individuals with SLI may be less likely than their TD peers to exhibit skilled prosocial behavior (Brinton, Fujiki, Montague, & Hanton, 2000; Fujiki, Brinton, Morgan, & Hart, 1999). Evidence from TD youths indicates that adolescents who perceive themselves as prosocial tend to be more socially confident and to spend more time in constructive peer activities (Jacobs, Vernon, & Eccles, 2004). The extent to which those with SLI regard themselves as prosocial remains in need of investigation.

Epidemiological studies have shown gender differences in the types of difficulties experienced by adolescents. Females are more likely to experience internalizing problems, for example, emotional difficulties, whilst males are more likely to exhibit externalizing disorders, such as conduct problems and hyperactivity (Rescorla et al., 2007). Research examining gender differences in adolescents with SLI is scant. There is some preliminary evidence that being a male adolescent with SLI is associated with conduct problems (Brownlie et al., 2004; Goh & O’Kearney, 2012). The typical female bias for emotional symptoms has not been observed in adolescents with SLI but evidence to date is based on a very small number of studies that include a small number of females (Conti-Ramsden & Botting, 2008; Wadman, Botting, Durkin, & Conti-Ramsden, 2011).

There is recognition in clinical and research work with young people with and without disabilities that the perceptions and views of individuals themselves are an important component of diagnosis, functioning and developmental prospects. In a longitudinal study of typical youth, Hoyt, Chase-Lansdale, McDade, and Adam (2012) reported that self-reported perceptions of wellbeing in adolescence, on measures such as feeling socially accepted, feeling loved, feeling just as good as others and enjoying life, predicted better perceived general health in young adulthood. Caldwell, Rudolph, Troop-Gordon, and Kim (2004) showed that negative self-views about relations with peers (e.g., “It's a waste of other kids’ time to be friends with me”) predicted social disengagement and stress, which in turn predicted subsequent social withdrawal and still more negative self-views. Thus, what young people perceive about their own social experiences and adjustment has the potential to fuel patterns of behavior and inferences that can become reciprocally self-perpetuating.

Currently, relatively little is known about the self-perceived problems of adolescents with SLI across a range of areas of their mental health. There is a need to determine how the young people see themselves and how they identify their own strengths and difficulties. In this investigation, we examine the social, emotional and behavioral functioning of 16-year-old adolescents with a history of SLI who are part of the Manchester Language Study. This study builds on the work carried out with this sample whose educational, cognitive and psychosocial functioning has been well documented (Botting & Conti-Ramsden, 2008; Conti-Ramsden, Durkin, Simkin, & Knox, 2009; Conti-Ramsden et al., 2012; St Clair et al., 2011).

In this investigation, we focus on adolescent self-reports for three key reasons. First, we wanted to determine the subjective perceptions of adolescents in respect of their strengths and difficulties. Second, while of interest in their own right as indicators of adjustment, subjective perceptions may also relate to variabilities in individual behavior and mental health (Walters, Stewart-Brown, & Fitzpatrick, 2003); for example, Litwack, Aikins, and Cillessen (2012) showed that adolescents’ perceptions of poor peer relations predicted depressive affect and lower self esteem, and Skaalvik (1997) found that adolescents who held negative self-perceptions tended to have higher levels of anxiety and poorer achievement orientation. Third, adolescents’ self-reported problems are strongly associated with mental health service need and utilization (Zwaanswijk, Van der Ende, Verhaak, Bensing, & Verhulst, 2003); furthermore, a high proportion of adolescents referred to clinical services have language impairments (Cohen, Farnia, & Im-Bolter, 2013). Thus, such self-reported data can provide clinically relevant information.

Our overall goals were twofold: first, we aimed to compare the two groups (those with a history of SLI versus TD adolescents) in respect of their perceptions of their social, emotional and behavioral functioning at age 16. Second, we wished to determine which factors predicted borderline or abnormal levels of strengths and difficulties. In respect of the first goal, previous evidence of greater difficulty in children with SLI led to the expectation that adolescents with a history of SLI would report more difficulties in emotional and behavioral functioning than do TD peers. In the social domain, we anticipated finding particular problems in peer relations but the research basis for the predictions for other aspects of social functioning – for example, prosocial behavior – was less clear. In respect of the second goal, we anticipated that gender would be associated with abnormal levels of difficulties (greater difficulties are frequently obtained among male samples, particularly in young people with developmental disorders). Of particular interest in respect of young people with SLI was the possible relationship between verbal and nonverbal skills and abnormal levels of difficulties in social, emotional and behavioral functioning.

## 2 Materials and methods

### 2.1 Participants

#### 2.1.1 Adolescents with a history of SLI

The young people were referred to as adolescents with a history of SLI, because they were originally part of a wider study: the Manchester Language Study (Conti-Ramsden & Botting, 1999; Conti-Ramsden, Crutchley, & Botting, 1997). The initial cohort of 242 children (6;6–7;9 years), which consisted of 186 boys (76.9%) and 56 girls (23.1%), were recruited from 118 language units across England and represented a random sample of 50% of all 7-year olds attending language units for at least half of the school week. Language units are specialized classes for children who have been identified with primary speech and language difficulties; the units are usually attached to mainstream schools. Children were excluded from the study if they were reported by their teachers as having frank neurological difficulties, hearing impairment, a diagnosis of autism or a general learning disability. All children had English as a first language. A small number, 27 (11.2%), had exposure to languages other than English at home. The children were contacted again at ages 8 (*N* = 232), 11 (*N* = 200), 14 (*N* = 113) and 16 (*N* = 139). The attrition observed was partly due to funding constraints at follow-up stages of the study. Participants retained for this stage were selected on the basis of traceability and geographical accessibility. Of the 139 young people (57.4% of the original cohort) who agreed to participate in the present stage of the study at age 16, 69.8% were males and 30.2% were females, ranging in age between 15;2 and 16;10 (*M* = 15;10; *SD* = 0.40 years). Of those who did not take part, contact has been lost with 51 individuals (21.1%) and 52 children (21.5%) did not consent to take part. There were no significant differences in the receptive, expressive and performance IQ (PIQ) standard scores at age 7 between those who participated at age 16 and those who did not, *p* > .3, nor were there significant differences in the distributions of household income and maternal education between the two groups, *p* > .5. There was, however, a significantly higher percentage of females amongst those who participated at age 16 than amongst those who did not, 30.2% vs 13.6%, respectively, *χ*^2^(1) = 9.19, *p* = .002.

Participants were classified as currently impaired if, at the time of the study, they met the following criteria for SLI: PIQ in the normal range (standard score of 85 or more) and concurrent receptive or expressive language standard scores of less than 85. Forty-eight participants (34.5%) were classified as SLI. Another 62 (44.6%) demonstrated concurrent impaired language as well as non-verbal skills, with PIQ and either receptive or expressive language scores below 85. It has been reported that some individuals with a history of SLI have declining PIQ across time (Conti-Ramsden et al., 2012). The changing SLI profiles of some of the participants were thus due to their PIQ scores having fallen since they were recruited to the study. There is evidence suggesting that children with low PIQ and language skills perform in important ways much like children with a history of SLI who have PIQ within the normal range (Leonard, 2003). At 16 years, the majority, 79.1%, of the respondents showed language ability in the impaired range. Twenty-three individuals (16.5%) had both receptive and expressive language standard scores in the normal range. The SLI status of the remaining 6 individuals could not be ascertained due to missing data. For simplicity, irrespective of their current SLI status, the 139 participants will be referred to as adolescents with a history of SLI throughout the paper.

#### 2.1.2 TD adolescents

A comparison group of 16-year-old adolescents from a broad background were recruited from the same schools as the participants with SLI, as well as targeted schools in selected demographic areas. All adolescents attending the schools and their families were contacted. The TD participants were selected to recruit a comparison group representative of households in England in terms of household income and maternal education level. Data from the General Household Survey were consulted (Office for National Statistics, 2001–2002). A total of 124 TD adolescents aged between 15;2 and 16;7 (*M* = 15;11; *SD* = 0.34 years) participated, of which 61.3% were males. These TD adolescents had no history of special educational needs or speech and language therapy provision.

All participants (SLI and TD) were attending their last year of compulsory secondary education. There was no significant difference in the sex ratio of males to females between the two groups, *χ*^2^(1) = 2.10, *p* = .15, or in the distribution of their household income, *χ*^2^(3) = 4.05, *p* = .26, or maternal education, *χ*^2^(2) = 1.85, *p* = .40. Comparisons of the psycholinguistic profiles of the adolescents with a history of SLI and their TD peers are shown in Table 1. Mean scores for the TD adolescents were all within the normal range. For the adolescents with a history of SLI, the average standard scores for receptive language and PIQ were around 1 *SD* below the population mean, whilst average expressive language was almost 2 *SD* below. As expected, one-way analyses of variance (ANOVA) showed that TD adolescents performed significantly better than their SLI counterparts in all three tests.

### 2.2 Measures

#### 2.2.1 Social, emotional and behavioral functioning

The self-report version of the Strengths and Difficulties Questionnaire (SDQ) was used. The SDQ is a 25 items behavioral questionnaire that can be administered to teachers, parents, and young people aged 11 or over (Goodman, 1997). The self-report version has been reported to discriminate satisfactorily between a community and mental health clinic sample of 11–16 years old (Goodman, Meltzer, & Bailey, 1998). Correlations between self-report SDQ scores and teacher- or parent-rated scores were also found to compare favorably with the average cross-informant correlations in previous studies of a range of measures (Goodman et al., 1998). Goodman et al. recommend that the SDQ could be used to assess young people's degree of awareness of their own problems. The 25 items of the SDQ are divided between 5 subscales: prosocial behavior, peer problems, emotional symptoms, conduct problems, and hyperactivity, with each subscale consisting of 5 items and each item being coded as ‘not true’, ‘somewhat true’ or ‘certainly true’. Scores for each of these subscales range from 0 to 10. For the prosocial subscale, for example, the 5 items include: ‘I try to be nice to other people’, ‘usually share with others’, ‘helpful if someone is hurt’, ‘kind to younger children’, and ‘often volunteer to help others’. For this subscale, the higher the rating the more prosocial the individual. In contrast, higher ratings in the other scales are associated with increased difficulties in these areas. Examples of items in the other subscales include: ‘Other children or young people pick on me or bully me’ (peer problems); ‘I worry a lot’ (emotional symptoms); ‘I get very angry and often lose my temper’ (conduct problems); ‘I am constantly fidgeting or squirming’ (hyperactivity). Thresholds for identifying normal, borderline, and abnormal behavior are also available for all the self-report subscales. The thresholds have been chosen so that roughly 80% of 11–16-year olds in the community are categorized as normal, 10% as borderline and 10% as abnormal. In this study we combined the ‘borderline’ and ‘abnormal’ categories.

#### 2.2.2 Language ability

Language skills were assessed using the Clinical Evaluation of Language Fundamentals-Revised (CELF-R; Semel, Wiig, & Secord, 1987). The Word Classes subtest was used to measure receptive language ability and the Recalling Sentences subtest was used to measure expressive language skills. These subtests were chosen as they are widely used in the literature and are considered good indicators of these skills (Conti-Ramsden, Botting, & Faragher, 2001; Gillon & Dodd, 2005; Stothard, Snowling, Bishop, Chipchase, & Kaplan, 1998).

#### 2.2.3 PIQ

PIQ was assessed using the full form of the UK version of the Wechsler Intelligence Scale for Children-Third Edition (WISC-III UK, Wechsler, 1992). This widely used assessment comprises the Block Design, Picture Completion, Coding, Picture Arrangement and Object Assembly subtests.

### 2.3 Procedure

Written informed consent was gained from all participants and ethical approval was gained from The University of Manchester. The adolescents were assessed and interviewed either at home or school on the above measures as part of a wider battery. Assessments took place in a quiet room with only the participant and a trained researcher present. Basic demographic information was collected and then the standardized assessments of PIQ and language were administered in the manner specified by the test manuals. For the SDQ, the items were read aloud to the participants. The items and response options were also presented visually. Care was taken to ensure that participants comprehended the items and the response options. Any inconsistent and unexpected responses were checked for meaning, particularly when the items were negatively worded, and extra clarification was given where needed. Very few interventions of this kind were required. For two-thirds of participants with a history of SLI, receptive and expressive language skills and PIQ were assessed at 14 years of age as funding constraints only allowed psycholinguistic tests to be conducted at age 16 if there were no data available from age 14 years.

### 2.4 Data analysis

All statistical analyses were conducted using Stata/SE 12.0 (StataCorp, 2011) and a two-tailed significance level of *p* = .05 was used. For each SDQ subscale, differences in the mean scores between adolescents with a history of SLI and TD adolescents were examined using ANOVA. Multivariate analysis of variance (MANOVA) was used to test for main effects of group and subscales by group interactions. For each of the SDQ subscale, binary logistic regression was used to examine which factors were associated with the odds of reporting borderline and abnormal behavior. The regression analyses were first carried out with all participants (adolescents with a history of SLI and their TD peers), to determine the differences between the two groups of individuals. The analyses were then repeated with adolescents with a history of SLI only.

## 3 Results

### 3.1 Social, emotional and behavioral functioning at age 16

Table 2 shows the SDQ subscale scores. Although the mean prosocial score for adolescents with a history of SLI was significantly lower than that of their TD peers, 7.8 (*SD* = 1.9) vs. 8.6 (*SD* = 1.5), *F*(1, 261) = 13.03, *p* < .001, partial *η*^2^ = 0.048, it was well within the range for typical prosocial behavior as reported in the SDQ (6–10). Overall, 13% of adolescents with a history of SLI compared with 4% of TD adolescents were found to display borderline/abnormal prosocial behavior. Similarly, adolescents with a history of SLI were more likely than their TD peers to report higher scores for hyperactivity [*M* = 4.5 (*SD* = 2.4) vs. *M* = 3.8 (*SD* = 2.3), *F*(1, 261) = 5.55, *p* = .019, partial *η*^2^ = 0.021], emotional symptoms [*M* = 3.8 (*SD* = 2.5) vs. *M* = 2.3 (*SD* = 1.9), *F*(1, 258) = 30.18, *p* < .001, partial *η*^2^ = 0.10], conduct problems [*M* = 2.5 (*SD* = 1.7) vs. *M* = 1.8 (*SD* = 1.6), *F*(1, 261) = 13.69, *p* < .001, partial *η*^2^ = 0.050] and peer problems [*M* = 2.5 (*SD* = 1.9) vs. *M* = 1.2 (*SD* = 1.1), *F*(1, 261) = 40.54, *p* < .001, partial *η*^2^ = 0.13]. MANOVA yielded a significant main effect of group, *F*(4, 255) = 14.43, *p* < .001, *η*^2^ = 0.18, whilst the difficulties subscales by group interaction was borderline significant, *F*(3, 256) = 2.54, *p* = .057. Of the four difficulties subscales, peer problems appeared to be the area showing the biggest difference between groups, with 26.6% of adolescents with a history of SLI being found to have borderline/abnormal levels of peer problems compared with 2.4% of their TD peers. There were no significant differences in SDQ scores by household income or maternal education.

### 3.2 Which factors are associated with reporting borderline and abnormal behavior?

#### 3.2.1 Adolescents with a history of SLI and TD individuals

For each of the SDQ domains, binary logistic regression analyses were conducted to examine which variables were associated with borderline or abnormally high levels of difficulties among the adolescents in this study (see Table 2). Outcomes were coded as ‘0’ for normal behavior (the reference category) and ‘1’ for borderline or abnormal behavior.

We investigated first the differences in strengths and difficulties between individuals with SLI and their TD counterparts. The regression models were built in two steps: PIQ standard scores and gender were entered first, followed by receptive language raw scores and group status, which was dummy coded with ‘0’ representing TD participants (the reference category) and ‘1’ the individuals with a history of SLI. Table 1 shows that while the mean language scores for the TD adolescents were within the normal range, for the adolescents with SLI, the average standard scores for receptive language were almost 1 *SD* below the population mean, whilst average expressive language was almost 2 *SD* below. Due to this large extent of non-overlap in the distributions of expressive language standard scores between the TD and SLI adolescents, expressive language was not investigated in these analyses which included group status as a covariate. Table 3 shows the odds ratios, standardized coefficients (change in odds for a *SD* increase in the explanatory variable) and their percentage equivalents for each of the models. With the exception of conduct problems, significant differences between SLI and TD adolescents in the odds of reporting borderline or abnormal levels of problems remained after controlling for gender, PIQ, and receptive language ability. The differential was particularly prominent for peer problems, with the odds of reporting borderline or abnormal level of difficulties being 12 times higher for SLI than for TD individuals.

Receptive language was a significant predictor in all the models except for peer problems. When the models were fitted additionally with two-way interaction terms of group status with gender and group status with receptive language, none of the interactions were significant, indicating that there was no evidence for differential gender or language effects on the odds of reporting abnormal behavior for individuals in the SLI and TD groups after controlling for the other covariates.

#### 3.2.2 Adolescents with a history of SLI only

The regression analyses were repeated with the SLI participants only. The models were built in two steps: PIQ standard scores and gender were entered first, followed by receptive and expressive language raw scores. The results are shown in Table 4. Among the individuals with a history of SLI, the estimated odds for males reporting borderline or abnormal prosocial behavior were 18 times higher than for females. Adolescents with better receptive language were also more likely to report borderline or abnormal prosocial behaviors. A 1 *SD* increase in the receptive language raw scores was associated with a 189% increase in the odds of reporting problems with prosocial behaviors. PIQ became significant only after controlling for gender, expressive and receptive language.

For hyperactivity, receptive language was the only significant predictor. Adolescents with a history of SLI with better receptive language were less likely to report problems with hyperactivity. This was also the case for emotional symptoms. PIQ became significant after controlling for gender, receptive and expressive language. A 1 *SD* increase in the PIQ standard scores was associated with a 68% increase in the odds of reporting emotional problems.

Gender, PIQ, receptive and expressive language abilities were not significant predictors of the odds of reporting borderline or abnormal levels of conduct or peer problems amongst adolescents with a history of SLI.

## 4 Discussion

The present study aimed to examine self-reported strengths and difficulties in social, emotional and behavioral functioning in a large sample of adolescents with a history of SLI. The findings raise a number of issues related to the mental health of these young people. First, as expected from previous research findings (Goh & O’Kearney, 2012; Im-Bolter & Cohen, 2007; Toppelberg & Shapiro, 2000), adolescents with a history of SLI reported more behavioral difficulties, more emotional symptoms, and more difficulties with peer relations than did their TD peers. These group differences were quite robust and most remained in the context of other influences such as gender, PIQ and language abilities. Difficulty with peer relations was the strongest differentiator between the groups. Longitudinal research with the same sample (St Clair et al., 2011), found evidence of increasing difficulties in peer relations from childhood to adolescence in these individuals with a history of SLI. This investigation adds to this body of research by determining that in adolescence, the odds of individuals with a history of SLI reporting clinical levels of difficulties in peer relations was 12 times higher than that of their TD peers. By 16 years of age, one in four adolescents with a history of SLI reported difficulties. This is in sharp contrast to the TD group, for whom peer relations were reported as an area of strength, with only 2.4% of adolescents reporting difficulties in this area. These findings dovetail with recent evidence that among samples of adolescents referred to clinics a substantial proportion have language impairment whereas non-referred comparison youths are much less likely to do so (Cohen et al., 2013).

Second, we examined the extent to which gender and language abilities predicted the likelihood of adolescents with SLI reporting borderline or abnormal levels of difficulties. Reports of behavioral difficulties included both hyperactivity traits such as ‘I am constantly fidgeting or squirming’ as well as problems with conduct, for example, ‘I get very angry and often lose my temper’. After adjusting for the effects of gender and nonverbal IQ, receptive language was a significant predictor of adolescent with a history of SLI reports of problems with hyperactivity and emotional symptoms. Receptive language was also a significant predictor when we included TD adolescents in the analysis. We found that the lower the ability of adolescents to understand spoken language, the more likely they would report having difficulties in these domains and with their conduct. Other aspects of language which were not measured in this study, for example, pragmatic language abilities, may also be implicated. This is an area that could fruitfully be addressed in future studies (see also Lindsay, Dockrell, & Strand, 2007).

Third, adolescents with a history of SLI did not perceive other aspects of social functioning as problematic. The majority of young people with a history of SLI reported prosocial attributes and thought that statements such as ‘I usually share with others’ applied to them. For approximately 87% of individuals with a history of SLI, this aspect of social functioning was a perceived strength. There were still overall group differences but it needs to be noted that the mean scores for prosocial behaviors were within the typical range for both groups. To the extent that self-perception as prosocial can be supportive of positive peer relations (cf. Jacobs et al., 2004), this is a relatively favorable finding. Given that participants with SLI do also perceive peer relations as difficult, it clearly does not mean that falling with the normal prosocial range will alone guarantee optimal outcomes. However, it does indicate an orientation toward other people that could be built upon by professionals working with this population. The small number of adolescents with a history of SLI who reported difficulties in this area were much more likely to be male. Regardless of gender, those who reported difficulties were also more likely to have better receptive language skills. The effect of receptive language was thus in the opposite direction than that observed in relation to problems in other areas of functioning such as emotional symptoms and hyperactivity. We speculate that in the context of positive behaviors, better receptive language may foster more awareness of difficulties. Given the lack of information on prosocial behaviors in adolescents with a history of SLI, however, the findings of this study are in need of further examination in future research.

Limitations of this study include the fact that, because of the practical and funding issues associated with a large scale longitudinal project, for two-thirds of participants with a history of SLI, it was not possible to measure language abilities contemporaneously with the SDQ at age 16 years. Instead, we used language measures collected at age 14 for these participants. It should be noted, however, that the most rapid changes in language development occur in early childhood (Gleason & Ratner, 2012) and that individual differences in language abilities tend to be stable over time (Beitchman et al., 1994; Bornstein, Hahn, & Haynes, 2004; Fernald & Marchman, 2012); during later childhood and into adolescence, in both typical and SLI populations, development occurs but is much less pronounced (Conti-Ramsden et al., 2012; Kemper, Rice, & Chen, 1995). Hence, language abilities at age 14 are good indicators of language abilities at age 16. Furthermore, because the language measures were collected in advance of the social and emotional measures, this provides a more dependable indication of any causal relationship between the former and the latter. Another limitation is that, while the study provides direct information on self-perceptions, we do not have corresponding data from independent informants, such as peers or parents. While it would certainly be advantageous in future research to triangulate evidence in this way, it remains important to understand how young people with developmental disabilities themselves perceive their own characteristics and their relations with others. This study contributes evidence from individuals, with a common but still relatively neglected disorder, whose voices are often unheard.

## 5 Conclusions

This study provides evidence that adolescents with a history of SLI perceive themselves as having social problems with peers and mental health difficulties. The findings have clinical implications for those working in speech language therapy, mental health and general practice. Data on the self-perceptions of adolescents with a history of SLI concerning their social, emotional and behavioral functioning gives relevant professionals useful information regarding these individuals’ awareness of their problems, their needs, and highlights likely traits that may lead to referral to services. It alerts those receiving referrals within CAMHS and community medicine services of the possible association between language impairment and mental health difficulties. Finally, it highlights the need to assess language abilities in adolescence, as this information has implications for the efficacy of verbally mediated interventions.

## Role of the funding sources

None of the funding sources have a role in the study design; in the collection, analysis and interpretation of data; in the writing of the report; and in the decision to submit the article for publication.

## Figures and Tables

**Table 1:** Participants’ psycholinguistic profiles (standard scores).

	Mean (*SD*)		One-way ANOVA	Partial *η*^2^
	SLI	TD		
Receptive language	83.8 (16.9)	99.5 (13.2)	*F*(1, 257) = 68.3, *p* < .001	0.21
Expressive language	73.9 (11.0)	97.2 (15.0)	*F*(1, 257) = 206.5, *p* < .001	0.45
PIQ	84.2 (18.7)	99.9 (15.8)	*F*(1, 255) = 52.9, *p* < .001	0.17

**Table 2:** Participants’ SDQ scores at age 16.

	Mean (*SD*)			% Reporting borderline/abnormal behavior
	Males	Females	Total	
Prosocial				
SLI	7.4 (2.0)	8.7 (1.4)	7.8 (1.9)	13.0
TD	8.3 (1.6)	9.0 (1.2)	8.6 (1.5)	4.0
Hyperactivity				
SLI	4.6 (2.4)	4.1 (2.4)	4.5 (2.4)	39.6
TD	3.4 (2.4)	4.3 (2.1)	3.8 (2.3)	20.2
Emotional				
SLI	3.5 (2.4)	4.6 (2.5)	3.8 (2.5)	28.3
TD	1.8 (1.4)	3.2 (2.1)	2.3 (1.9)	6.6
Conduct				
SLI	2.6 (1.8)	2.3 (1.7)	2.5 (1.7)	25.2
TD	1.9 (1.8)	1.5 (1.4)	1.8 (1.6)	12.9
Peer				
SLI	2.6 (1.9)	2.3 (1.8)	2.5 (1.9)	26.6
TD	1.3 (1.1)	1.2 (1.2)	1.2 (1.1)	2.4

**Table 3:** Binary logistic regression analyses predicting the odds of reporting borderline or abnormal behavior, comparing adolescents with a history of SLI and TD individuals.

SDQ scale	Variable	Odds ratio	95% CI	Change in odds for *SD* increase in variable	% Change in odds for *SD* increase in variable
Prosocial (*N* = 257)					
Step 1	Gender (ref: female)	6.77*	1.52–30.2	–	–
	PIQ	0.97**	0.94–0.99	0.52	–48
Step 2	Gender (ref: female)	8.34**	1.78–39.0	–	–
	PIQ	0.95**	0.92–0.98	0.37	−63
	Receptive language	1.21*	1.04–1.40	2.40	140
	Group status (ref: TD)	3.38*	1.05–10.8	–	–
Hyperactivity (*N* = 257)					
Step 1	Gender (ref: female)	1.58	0.88–2.84	–	–
	PIQ	0.99	0.98–1.00	0.82	−18
Step 2	Gender (ref: female)	1.28	0.70–2.36	–	–
	PIQ	1.01	0.99–1.03	1.26	26
	Receptive language	0.90**	0.84–0.97	0.62	−38
	Group status (ref: TD)	2.04*	1.06–3.93	–	–
Emotional (*N* = 254)					
Step 1	Gender (ref: female)	0.52	0.27–1.00	–	–
	PIQ	0.99	0.97–1.01	0.80	−20
Step 2	Gender (ref: female)	0.32**	0.15–0.68	–	–
	PIQ	1.02	1.00–1.05	1.53	53
	Receptive language	0.90*	0.82–0.98	0.61	−39
	Group status (ref: TD)	5.92**	2.32–15.1	–	–
Conduct (*N* = 257)					
Step 1	Gender (ref: female)	1.74	0.86–3.52	–	–
	PIQ	0.98*	0.96–1.00	0.69	−31
Step 2	Gender (ref: female)	1.50	0.73–3.08	–	–
	PIQ	1.00	0.98–1.02	0.94	−6
	Receptive language	0.92*	0.85–1.00	0.68	−32
	Group status (ref: TD)	1.42	0.65–3.09	–	–
Peer problems (*N* = 257)					
Step 1	Gender (ref: female)	1.27	0.60–2.71	–	–
	PIQ	0.97**	0.95–0.99	0.58	−42
Step 2	Gender (ref: female)	0.98	0.44–2.19	–	–
	PIQ	0.99	0.97–1.02	0.90	−10
	Receptive language	0.98	0.90–1.07	0.90	−10
	Group status (ref: TD)	12.0**	3.35–42.8	–	–

PIQ: standard scores used; receptive language: raw scores used.

* *p* < .05.

** *p* < .01.

**Table 4:** Binary logistic regression analyses predicting the odds of reporting borderline or abnormal behavior in adolescents with a history of SLI.

SDQ scale	Variable	Odds ratio	95% CI	Change in odds for *SD* increase in variable	% Change in odds for *SD* increase in variable
Prosocial (*N* = 133)					
Step 1	Gender (ref: female)	9.67*	1.21–77.1	–	–
	PIQ	0.98	0.95–1.01	0.63	−37
Step 2	Gender (ref: female)	18.0*	2.00–162.1	–	–
	PIQ	0.93**	0.89–0.98	0.28	−72
	Receptive language	1.23*	1.02–1.50	2.89	189
	Expressive language	1.05	0.98–1.11	1.84	84
Hyperactivity (*N* = 133)					
Step 1	Gender (ref: female)	1.61	0.73–3.53	–	–
	PIQ	1.00	0.98–1.02	1.06	6
Step 2	Gender (ref: female)	1.47	0.65–3.30	–	–
	PIQ	1.02	1.00–1.04	1.46	46
	Receptive language	0.86**	0.78–0.96	0.48	−52
	Expressive language	1.02	0.98–1.05	1.27	28
Emotional (*N* = 132)					
Step 1	Gender (ref: female)	0.62	0.27–1.39	–	–
	PIQ	1.01	0.99–1.03	1.18	19
Step 2	Gender (ref: female)	0.53	0.23–1.24	–	–
	PIQ	1.03*	1.00–1.05	1.68	68
	Receptive language	0.87*	0.78–0.97	0.50	−50
	Expressive language	1.01	0.97–1.05	1.15	15
Conduct (*N* = 133)					
Step 1	Gender (ref: female)	1.35	0.56–3.26	–	–
	PIQ	0.99	0.97–1.01	0.84	−16
Step 2	Gender (ref: female)	1.25	0.51–3.08	–	–
	PIQ	1.00	0.98–1.03	1.04	4
	Receptive language	0.92	0.83–1.02	0.66	−34
	Expressive language	1.00	0.97–1.04	1.03	4
Peer problems (*N* = 133)					
Step 1	Gender (ref: female)	1.15	0.49–2.72	–	–
	PIQ	0.99	0.97–1.01	0.88	−12
Step 2	Gender (ref: female)	1.17	0.49–2.82	–	–
	PIQ	1.00	0.97–1.02	0.96	−4
	Receptive language	1.02	0.92–1.13	1.10	10
	Expressive language	0.97	0.94–1.01	0.68	−32

PIQ: standard scores used; receptive and expressive language: raw scores used.

* *p* < .05.

** *p* < .01.
